# Hepatitis A-Induced Fulminant Hepatic Failure Linked to Autoimmune Hepatitis in a Child Amidst Diagnostic Challenges of Dengue and Malaria

**DOI:** 10.7759/cureus.89310

**Published:** 2025-08-03

**Authors:** Muhammad Bilal, Muhammad Usman Ajmal, Ahtisham Ali

**Affiliations:** 1 Paediatrics, Farooq Hospital, West Wood Branch, Lahore, Lahore, PAK; 2 Paediatrics, University of Child Health Sciences and The Children’s Hospital, Lahore, Lahore, PAK

**Keywords:** autoimmune hepatitis, case report, dengue, fulminant hepatic failure, hepatitis a, malaria, paediatrics

## Abstract

Hepatitis A virus (HAV) typically causes a self-limiting illness in children. Rarely, it can progress to fulminant hepatic failure (FHF), and even less commonly, may be followed by features suggestive of autoimmune hepatitis (AIH). The diagnostic overlap can be particularly challenging in tropical regions, where endemic infections such as dengue and malaria may present with similar clinical features.

We report a previously healthy five-year-old girl who presented with a four-day history of high-grade fever, abdominal pain, vomiting and lethargy. Examination revealed pallor, icterus, hepatosplenomegaly, and right-sided pleural effusion. Initial investigations showed thrombocytopenia, leukopenia, and ascites, prompting a provisional diagnosis of dengue hemorrhagic fever. However, dengue serology was negative. Further workup revealed HAV IgM positivity and Plasmodium vivax parasitemia. Her condition worsened with bilateral pleural effusions and altered mental status, consistent with mild hepatic encephalopathy. She was managed conservatively without liver transplantation using intravenous antibiotics, oral antimalarials, lactulose, rifaximin, and transfusional support. Clinical stabilisation was achieved, and she was discharged. Persistent transaminitis on follow-up prompted further evaluation, revealing antinuclear antibody (ANA) positivity (1:320, speckled), suggestive of evolving type 1 AIH. Oral corticosteroid therapy was initiated, leading to a favourable response. She continues on outpatient follow-up with improving liver function.

While definitive confirmation of AIH was limited by the absence of serum IgG, smooth muscle antibody (SMA), and liver biopsy, the findings were suggestive of an evolving autoimmune process. This case highlights the diagnostic pitfalls in co-endemic regions and underscores the importance of re-evaluating children with persistently elevated liver enzymes following acute viral hepatitis.

## Introduction

Fulminant hepatic failure (FHF) in children is a life-threatening condition characterised by the rapid onset of liver dysfunction, coagulopathy, and encephalopathy in the absence of pre-existing chronic liver disease [1]. While viral hepatitis, especially hepatitis A, is among the most common causes in resource-limited countries [2], autoimmune and metabolic conditions must be considered, particularly in children who fail to recover as expected or present with atypical features [3].

Hepatitis A virus (HAV) typically causes a self-limiting illness in children, but in some cases, it can result in acute liver injury or even FHF [4]. In Pakistan, a recent hospital-based study reported that HAV accounted for up to 75% of acute viral hepatitis cases in children [5]. Diagnosing such cases can be challenging in tropical regions, where other endemic infections, such as dengue and malaria, can present with overlapping symptoms, including fever, jaundice, hepatosplenomegaly, thrombocytopenia, ascites, and pleural effusion [6]. In resource-limited settings, early management often focuses on the most probable infectious etiologies, which may delay the diagnosis of underlying or co-existing immune-mediated conditions.

Autoimmune hepatitis (AIH), an inflammatory disease of the liver of unknown aetiology, is rare in children, but remains an important differential diagnosis in unexplained or prolonged hepatitis. It is commonly associated with positive autoimmune markers, such as antinuclear antibody (ANA) or anti-liver kidney microsomal (LKM) antibodies, but may also present with seronegative profiles initially [7]. Viral infections, including HAV, have been proposed as possible triggers for the first manifestation of AIH [8]. One proposed mechanism is molecular mimicry, where viral antigens resemble host liver proteins, potentially initiating an autoimmune response in genetically susceptible individuals.

This case report describes a child who presented with clinical and biochemical features suggestive of dengue hemorrhagic fever, but was later found to have HAV infection with co-existing plasmodium vivax parasitemia. Her condition progressed to FHF, for which she was managed conservatively and discharged in stable condition. However, on follow-up, persistently elevated liver enzymes and serological findings raised the suspicion of autoimmune hepatitis, and she is currently being managed on oral corticosteroid therapy in an outpatient setting. This case highlights the diagnostic complexity of hepatic presentations in tropical populations and underscores the need for re-evaluation when liver function fails to normalise after viral hepatitis.

## Case presentation

A previously healthy five-year-old girl was admitted to the paediatric high dependency unit of a tertiary care hospital in Lahore, Pakistan, with a four-day history of high-grade, intermittent fever associated with rigours, chills, abdominal pain, and vomiting.

Initial examination revealed her blood pressure at 100/55 mmHg, pulse rate at 130 beats/min, respiratory rate at 28 breaths/min, temperature at 99°F, and oxygen saturation at 98% on room air. Her random blood glucose was 90 mg/dL (normal: 70-110 mg/dL). She was conscious and well-oriented but appeared lethargic, pale, and had clinical icterus. No cyanosis, clubbing, lymphadenopathy, or oedema was noted. Chest auscultation revealed reduced air entry on the right side. The abdomen was soft and tender at the right hypochondrium with hepatosplenomegaly. Initial laboratory investigations are shown in Table 1. 

**Table 1:** Baseline laboratory investigations at admission Initial leukopenia and thrombocytopenia raised suspicion for dengue, later ruled out serologically. CRP: C-reactive protein

Parameter	Value	Normal Range (SI)
Hemoglobin	10.6 g/dL	11.5–15.5 g/dL
Total Leukocyte Count	3.9 ×10⁹/L	4.5–13.5 ×10⁹/L
Neutrophils	66%	40–75%
Lymphocytes	29%	20–40%
Platelets	40 ×10⁹/L	150–400 ×10⁹/L
CRP	87.2 mg/L	<5 mg/L

**Figure 1:**
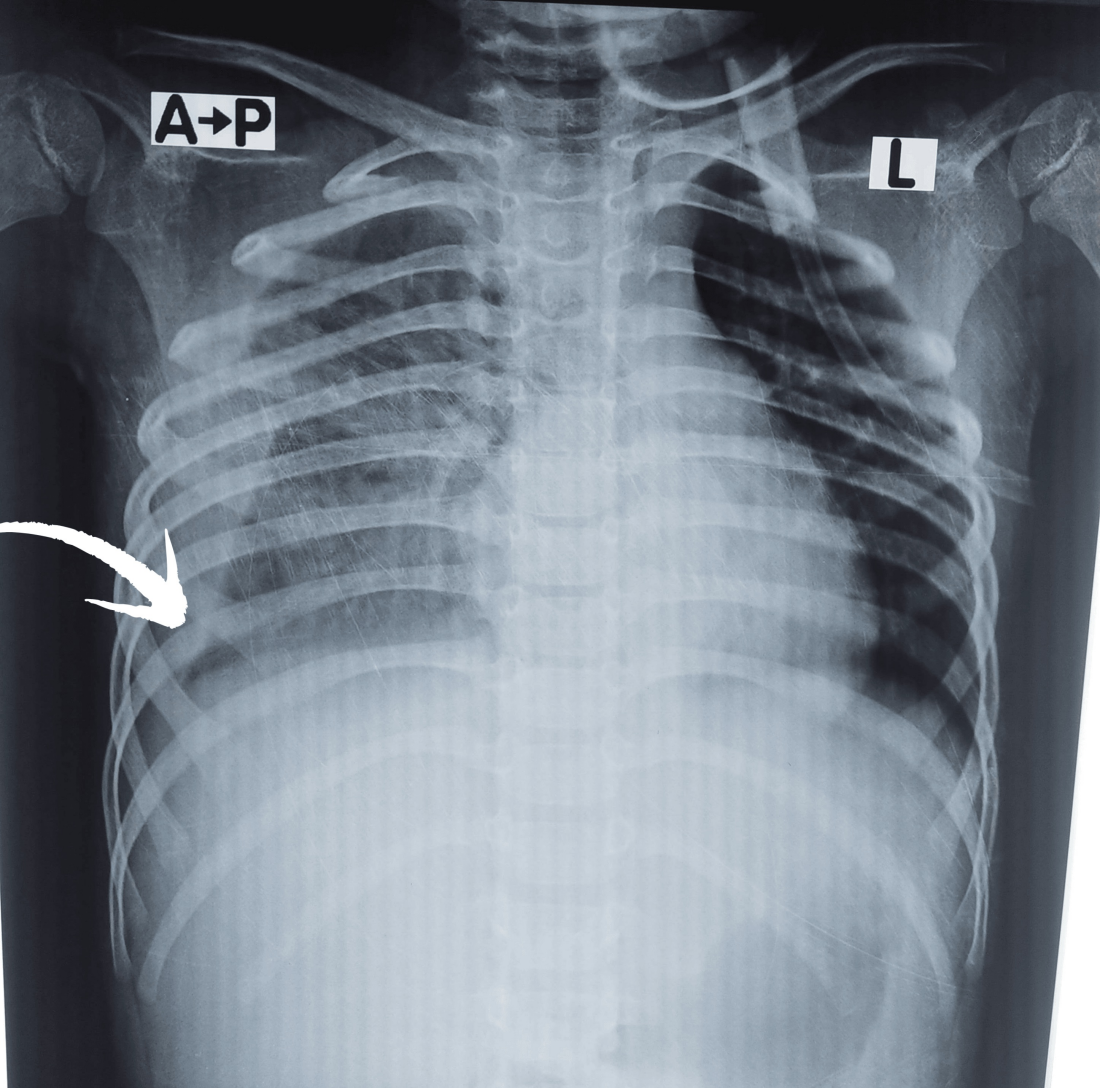
Chest radiograph demonstrating moderate right-sided pleural pleural effusion (~250 mL). Note the blunting of the right costophrenic angle and homogeneous opacity in the lower lung zone.

Ultrasound abdomen showed hepatosplenomegaly with mild ascites. The liver was moderately enlarged with slightly increased echogenicity, smooth surface, and regular margins, without any focal lesions. Chest X-ray revealed right-sided pleural effusion as shown in Figure 1. Considering thrombocytopenia and plasma leak features, and given the patient's residence in a dengue-endemic and mosquito-prevalent area, a provisional diagnosis of dengue hemorrhagic fever (DHF) was made. NS1 antigen and IgM were sent, and the child was managed with the local protocols.

After 12 hours, both NS1 and dengue IgM came back negative, prompting re-evaluation. A paediatric gastroenterologist was consulted and additional labs were sent, including peripheral blood smear, liver function tests, renal function tests, serum electrolytes, coagulation profile, serum amylase, lipase and hepatitis viral markers. Repeat Investigations are summarised in Table 2.

**Table 2:** Laboratory investigations after re-evaluation Marked transaminitis and INR elevation consistent with hepatocellular injury and early hepatic dysfunction. ALT: alanine aminotransferase, AST: aspartate aminotransferase, ALP: alkaline phosphatase, PT: prothrombin time, INR: international normalised ratio, APPT: activated partial thromboplastin time

Parameter	Value	Normal Range
ALT	1430 U/L	<40 U/L
AST	2250 U/L	<40 U/L
ALP	512 U/L	80–360 U/L
Total bilirubin	8.1 mg/dL	0.3–1.2 mg/dL
Albumin	2.9 g/dL	3.5–5.0 g/dL
PT	21.5 sec	11–14 sec
INR	1.9	0.9–1.2
APTT	39.3 sec	25–35 sec
Urea	15 mg/dL	10–40 mg/dL
Creatinine	0.4 mg/dL	0.3–0.7 mg/dL
Serum Sodium	133 mmol/L	135–145 mmol/L
Serum potassium	4.8 mmol/L	3.5–5.0 mmol/L
Amylase	29 U/L	28–100 U/L
Lipase	35 U/L	13–60 U/L

Serological testing revealed hepatitis A IgM positivity, confirming acute hepatitis A-induced FHF. Peripheral blood smear was also positive for Plasmodium vivax, supporting concurrent malarial parasitemia. While the level of parasitemia was not quantified, the diagnosis of malaria was established based on the presence of Plasmodium vivax trophozoites identified on peripheral blood smear. Over 24 hours, the child became tachypneic, requiring 4L/min oxygen. Repeat ultrasound showed increased ascites, hepatosplenomegaly, increased right-sided pleural effusion (250ml) and a new left-sided pleural effusion (70ml) as well. She also developed altered mental status, signalling mild hepatic encephalopathy.

Initially, a conservative approach was adopted; however, following a consultation with gastroenterology, a diagnostic and therapeutic ultrasound-guided pleural tap was performed. 220 mL of exudative fluid was drained. Analysis showed: protein 4.7 mg/dL, albumin 3.0 g/dL, lactate dehydrogenase (LDH) 476 U/L, glucose 78 mg/dL, WBC 1970/mm³ (80% lymphocytes), RBC 3880/mm³. Culture and acid-fast bacillus (AFB) stain were negative. Although the pleural fluid albumin level (3.0 g/dL) was slightly higher than the concurrent serum albumin (2.9 g/dL), this minor discrepancy may reflect laboratory variability or timing differences in sample collection and was not deemed clinically significant. 

Management included intravenous fluids, empirical intravenous antibiotics, fresh frozen plasma, and albumin infusions to address coagulopathy and hypoalbuminemia. She was also started on oral rifaximin and lactulose for encephalopathy, along with spironolactone and ursodeoxycholic acid for ascites and cholestasis. She also received a seven-day course of oral artemether-lumefantrine.

The patient underwent a 12-day inpatient course. Additional tests such as ceruloplasmin, anti-LKM antibodies, ANA, and pleural adenosine deaminase (ADA) levels were within normal ranges, ruling out alternative causes of hepatic failure. Drug-induced liver injury was also considered but deemed unlikely, as there was no known exposure to hepatotoxic medications. An ultrasound before discharge showed complete resolution of ascites and left-sided pleural effusion with only mild residual right-sided effusion remaining. Discharge investigations are provided in Table 3.

**Table 3:** Laboratory investigations at discharge Persistent hyperbilirubinemia despite clinical improvement may indicate delayed resolution of hepatic inflammation or cholestasis. TLC: total leukocyte count, AST: aspartate aminotransferase, ALT: alanine aminotransferase, PT: prothrombin time, INR: international normalised ratio, APPT: activated partial thromboplastin time

Parameter	Value	Normal Range
Hemoglobin	10.4 g/dL	11.5–15.5 g/dL
Platelets	179 ×10⁹/L	150–400 ×10⁹/L
TLC	8.4 ×10⁹/L	4.5–13.5 ×10⁹/L
Neutrophils	42%	40–75%
Lymphocytes	46%	20–40%
Sodium	137 mmol/L	135–145 mmol/L
Potassium	4.1 mmol/L	3.5–5.0 mmol/L
AST	162 U/L	<40 U/L
ALT	393 U/L	<40 U/L
Bilirubin	12.4 mg/dL	0.3–1.2 mg/dL
Albumin	4.3 g/dL	3.5–5.0 g/dL
PT	12 sec	11–14 sec
INR	1.0	0.9–1.2
APTT	32.9 sec	25–35 sec

**Figure 2:**
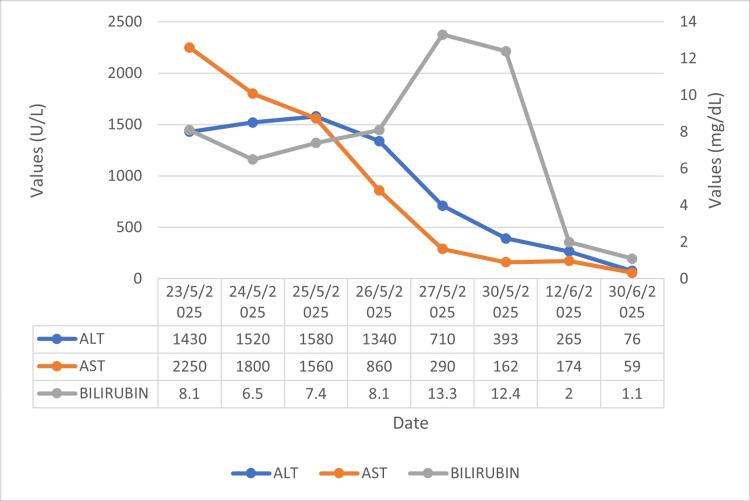
Serial trends in ALT, AST, and total bilirubin from admission through follow-up. ALT and AST peaked early and declined progressively. Total bilirubin peaked later and normalized during follow-up. ALT: alanine aminotransferase, AST: aspartate aminotransferase

Two weeks after discharge, at her first follow-up visit, the patient was clinically well but had persistently elevated transaminases (alanine aminotransferase (ALT): 265 U/L, aspartate aminotransferase (AST): 174 U/L), prompting further evaluation. Repeat testing revealed a positive ANA at a titer of 1:320 with a speckled pattern. These findings raised strong suspicion of evolving type 1 autoimmune hepatitis. Serum immunoglobulin G (IgG), anti-smooth muscle antibody (SMA), and liver biopsy could not be performed due to test unavailability and parental refusal. Based on the clinical course and laboratory findings, oral prednisolone was initiated at a dose of 2 mg/kg/day on day 14 post-discharge. Over the following weeks, the patient showed steady clinical improvement, with a significant decline in liver enzyme levels on serial follow-up testing, consistent with a favourable response to corticosteroid therapy. She continues to be monitored on an outpatient basis. Figure 2 illustrates the serial trends in ALT, AST, and total bilirubin across admission, hospital stay, and post-discharge follow-up, showing gradual normalisation of transaminases and delayed bilirubin clearance.

## Discussion

FHF in children is a rare but critical condition requiring prompt diagnosis and specialised care. In tropical regions, early clinical suspicion often leans toward endemic infectious diseases such as dengue hemorrhagic fever, especially when features like fever, thrombocytopenia, pleural effusions, and ascites are present [1,6]. However, hepatitis A virus, though usually self-limiting in children, remains a leading cause of FHF in these settings [2].

In our case, the initial clinical picture mimicked DHF, a common pitfall in South Asia due to overlapping features such as jaundice, hepatomegaly and elevated liver enzymes. While the incidence of acute liver failure in DHF is very low, it is associated with a higher mortality rate [6]. The presence of Plasmodium vivax parasitemia further supported a presumptive vector-borne infection. However, negative dengue markers and a positive HAV IgM shifted the diagnostic focus. This is consistent with findings by Talat et al., who identified hepatitis A as the most common aetiology of paediatric FHF in their tertiary care cohort in Pakistan [2].

Although HAV is typically benign, cases progressing to FHF are well-documented. Gautam et al. recently reported favourable outcomes in children with HAV-induced liver failure, including native liver survival despite severe presentations [4]. Our patient followed a similar clinical course, with encephalopathy and decompensation managed conservatively without transplantation.

Persistent elevation of transaminases after discharge prompted further autoimmune evaluation, revealing a positive ANA at a titer of 1:320 with a speckled pattern. This clinical evolution was suggestive of AIH. However, the diagnosis of autoimmune hepatitis in this case remains presumptive. Serum IgG levels and anti-SMA testing were not available at our centre, and liver biopsy was deferred due to parental refusal. As a result, the International Autoimmune Hepatitis Group (IAIHG) scoring system could not be applied due to incomplete data. Mendez-Sanchez and Pal have postulated HAV as a potential trigger for AIH, possibly through immune-mediated mechanisms like molecular mimicry [3]. While other viral infections like Epstein-Barr virus (EBV), Cytomegalovirus (CMV), and Hepatitis E virus (HEV) have also been identified as possible triggers for the first manifestation of AIH in adults [8], HAV may present a similar post-viral association. 

Autoimmune hepatitis in paediatric patients can be seronegative at onset or present solely with ANA positivity. Islek and Keskin emphasised that seronegative AIH in children is not uncommon [7], so a high index of suspicion must be maintained when liver function fails to normalise despite clinical recovery [9]. In our patient, the initiation of corticosteroid therapy led to biochemical improvement, reinforcing the diagnosis.

This case underscores the diagnostic complexity of hepatic presentations in co-endemic tropical regions and highlights the importance of reassessing children who fail to show biochemical recovery after acute viral hepatitis. While the diagnosis of autoimmune hepatitis in this patient remains presumptive, the clinical evolution and response to corticosteroid therapy raise the possibility of a post-viral autoimmune process. Early recognition of post-viral AIH and timely corticosteroid initiation can be crucial in altering long-term outcomes [7,9].

## Conclusions

This case highlights a rare but important instance where HAV-induced FHF may have precipitated features suggestive of autoimmune hepatitis. It further emphasises the diagnostic pitfalls in areas where multiple infections such as dengue and malaria co-exist, and the need for re-evaluating children with persistent transaminitis following an acute viral illness.
